# Supplementary material for: Assessment of aerosol persistence in ICUs via low-cost sensor network and zonal models
Source: Sci Rep. 2023 Mar 10;13:3992. doi: 10.1038/s41598-023-30778-7 (PMC10006437; doi:10.1038/s41598-023-30778-7)
Supplement: Supplementary file 1 — Supplementary Information. [file 41598_2023_30778_MOESM1_ESM.pdf]

Supplementary Information

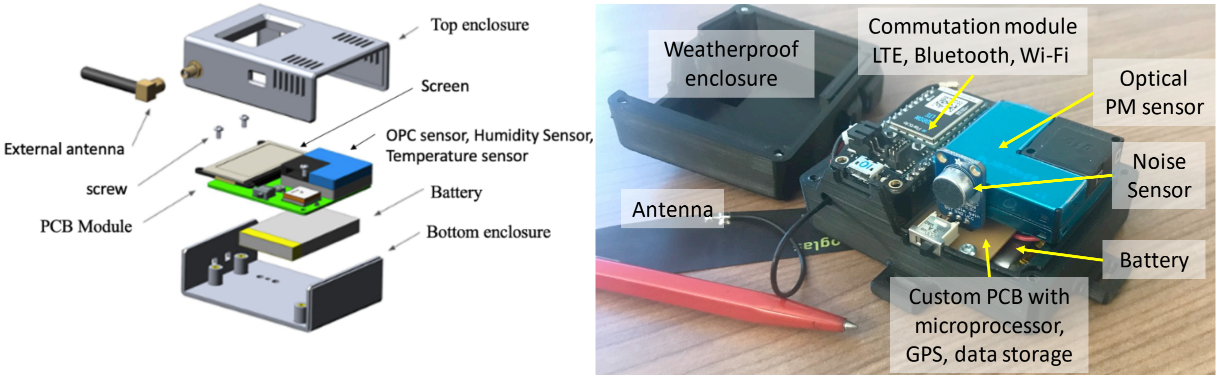

Supplementary Figure 1: Left: Exploded view of the AeroSpec sensors used for the study consisting of PCB board, LiPo battery, and display. The electronics are assembled in an ABS plastic enclosure, with dimensions of (H) 100mm x (W) 60mm x (D) 25mm and a weight of 120g. Right: IoT-enabled Aerosol Sensors The cost of the components is ~US100$.

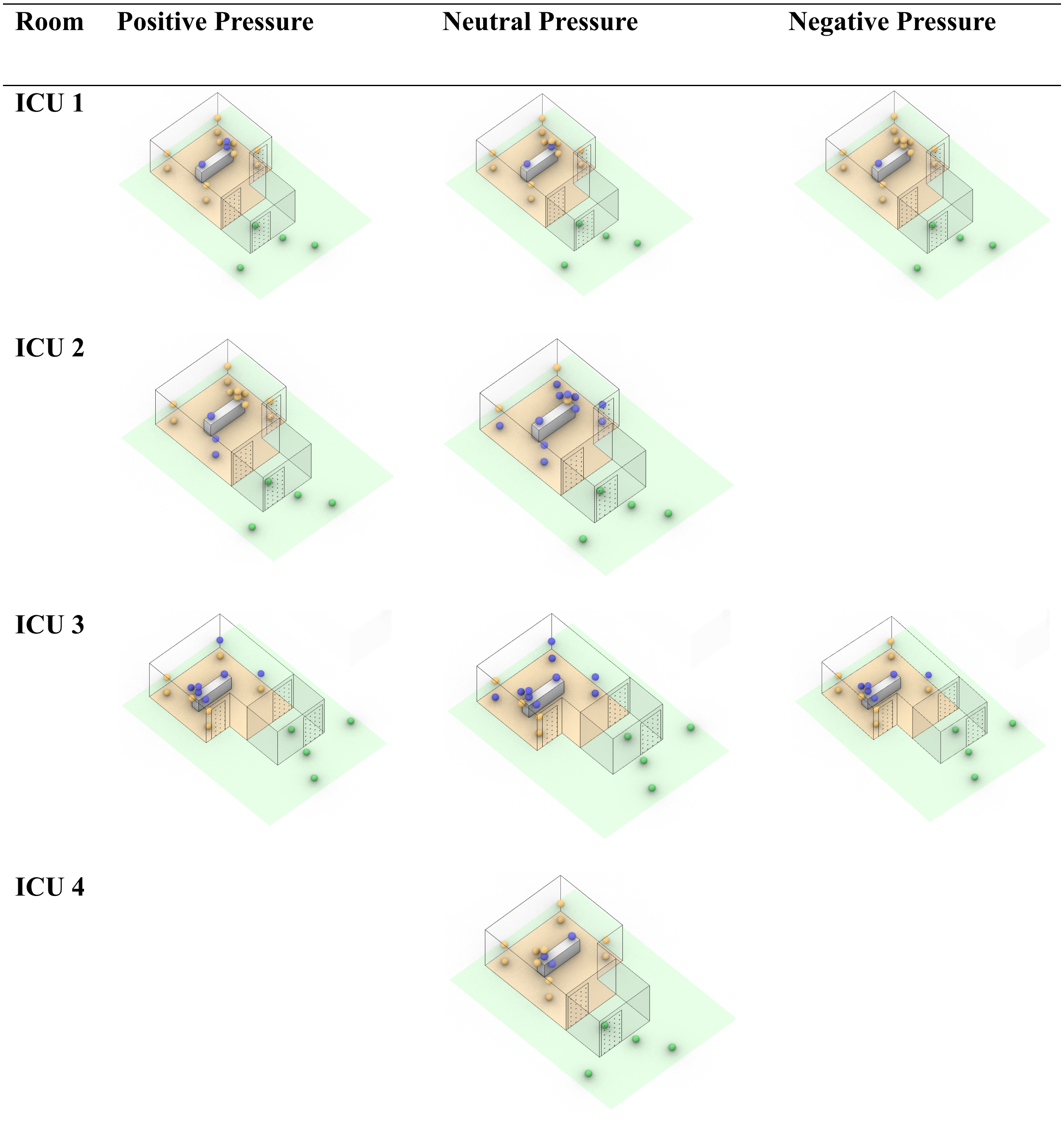

Supplementary Figure 2: Automated zones created by the k-means clustering algorithm for each experiment. Blue sensors represent Zone 1, orange sensors represent Zone 2, and green sensors represent Zone 3.

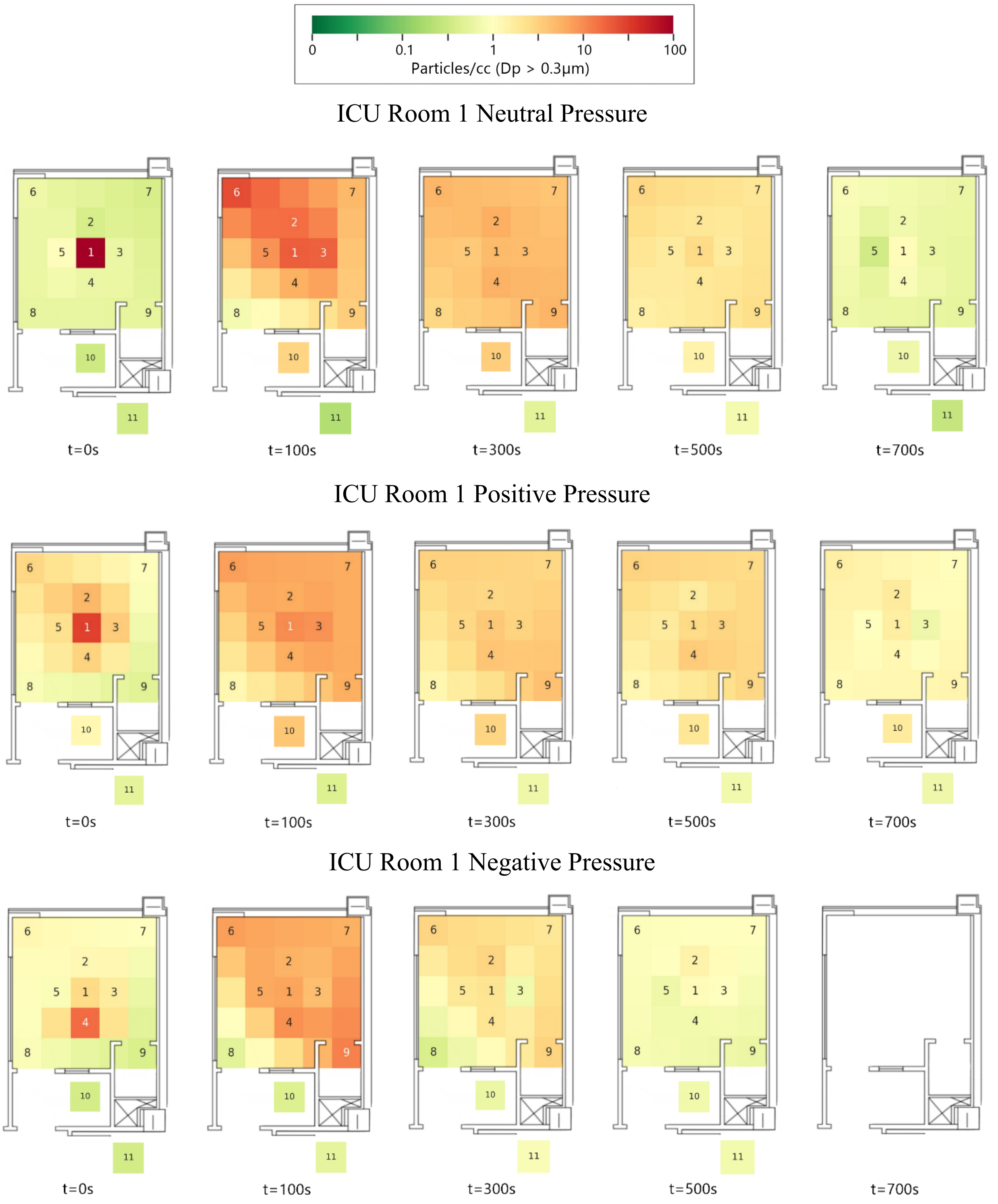

Supplementary Figure 3: 2D heat maps of ICU room 1 averaged across three experiments for each case of (top) Neutral pressure, (middle) Positive pressure, and (bottom) Negative pressure.

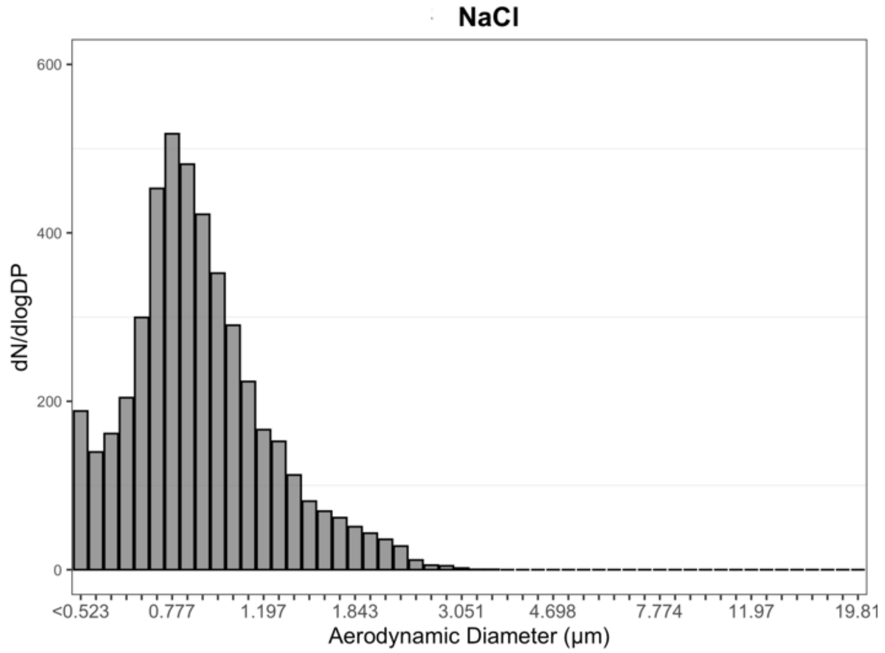

Supplementary Figure 4: Normalized particle NaCl after nebulization was measured by the Aerodynamic Particle Sizer (TSI APS3321) in aerosol chamber experiments; the chamber design is described in refs [85, 86] The median diameter is 0.86 µm.

Supplementary Table 1: ICU room dimensions, number, air vents dimensions and measured flow velocity exhaust vents and door gaps. ICU 1 and 2 measurements were not taken, they both were occupied on the day when these measurements were taken.

Room	Dim (ft)	Door		Vents/ Air Intakes	
		Flow Velocity (m/s)	Dim (ft^2)	Flow Velocity (m/s)	Dim (ft^2)
ICU 3	15x17	2.67	0.12	N/A	N/A
ICU 4	16x16	0.42	0.12	0.45	1
